# Recent Progress in Electrocatalysts for Hydroquinone Electrochemical Sensing Application

**DOI:** 10.3390/bios15080488

**Published:** 2025-07-28

**Authors:** Mohammad Aslam, Khursheed Ahmad, Saood Ali, Khaled Hamdy, Danishuddin

**Affiliations:** 1 School of Chemical Engineering, Yeungnam University, Gyeongsan 38541, Republic of Korea; 2 Discipline of Chemistry, Indian Institute of Technology, Indore 453552, India; 3 School of Mechanical Engineering, Yeungnam University, Gyeongsan 38541, Republic of Korea; 4 Department of Biotechnology, Yeungnam University, Gyeongsan 38541, Republic of Korea

**Keywords:** hydroquinone, electrochemical sensors, biosensors, composites

## Abstract

This review article compiled previous reports in the fabrication of hydroquinone (HQ) electrochemical sensors using differently modified electrodes. The electrode materials, which are also called electrocatalysts, play a crucial role in electrochemical detection of biomolecules and toxic substances. Metal oxides, MXenes, carbon-based materials such as reduced graphene oxide (rGO), carbon nanotubes (CNTs), layered double hydroxides (LDH), metal sulfides, and hybrid composites were extensively utilized in the fabrication of HQ sensors. The electrochemical performance, including limit of detection, linearity, sensitivity, selectivity, stability, reproducibility, repeatability, and recovery for real-time sensing of the HQ sensors have been discussed. The limitations, challenges, and future directions are also discussed in the conclusion section. It is believed that the present review article may benefit researchers who are involved in the development of HQ sensors and catalyst preparation for electrochemical sensing of other toxic substances.

## 1. Introduction

The monitoring of environmental pollutants is of great significance to reduce environmental pollution [1]. Environmental pollution is one of the major threats for the future world [1]. There are various phenolic compounds which are widely used in various industries and released to the environment in the form of waste water or industrial effluents [2]. In particular, hydroquinone (HQ) is one of the aromatic compounds which belongs to the phenolic family [3]. HQ is widely used in various industries such as dye, cosmetics paper production, etc. [4,5]. It is understood that prolonged exposure to HQ may lead to adverse health issues on human health such as fatigue and kidney damage [4]. The HQ also possesses negative impacts on environment and aquatic life [5]. The monitoring of HQ is necessary for human welfare and the environment. In this regard, conventional methods such as high-performance liquid chromatography (HPLC) [6], fluorescence [7], chemiluminescence [8], flow injection [9], and solid-phase extraction [10] were utilized for the determination of HQ. Unfortunately, conventional methods require complex laboratory setups, high-cost equipment, and highly skilled operators [11]. In recent years, electrochemical sensing technology has received enormous interest from the scientific community for the determination of environmental pollutants [12]. Electrochemical technology has several advantages such as high selectivity, simplicity, real-time detection in real samples, repeatability, reproducibility, being cost-effective, and stability [13]. Electrode materials, which are also known as electrocatalysts, play a vital role in improving sensing performance of electrochemical sensors [14]. Electrode materials with larger surface area, high porosity, high electrical conductivity, and electrochemical properties are desirable materials for electrochemical applications [15]. In previous years, various electrocatalysts such as metal oxides [16], polymers [17], carbon-based materials [18], metal sulfides [19], and hybrid composites [20] were used for electrochemical sensing applications. It was believed from the previous studies that the electrochemical sensing technique is a promising approach for the monitoring of HQ.

This review article aims to summarize the recent development in HQ sensing with specific focus on the preparation of electrocatalyst materials (metal oxides, rGO, MXenes, LDH, metal sulfides, and hybrid composites) for the modification of working electrodes. This review also highlights the sensing performance and application of fabricated electrodes in real sample studies of HQ. We believe that the present work would be useful for the electrochemists and materials scientists who are developing HQ sensors.

## 2. HQ Electrochemical Sensors

**Scheme 1:**
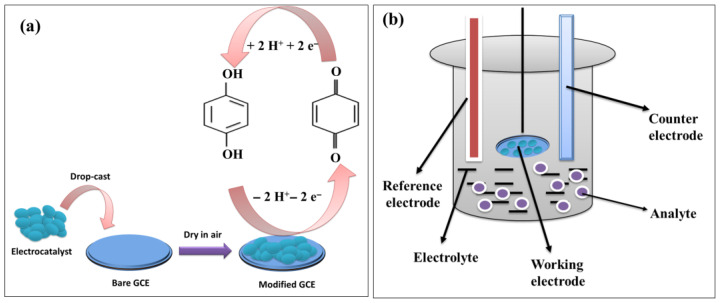
Schematic illustration of the (**a**) fabrication of GCE for HQ sensing and (**b**) three-electrode assembly for electrochemical detection process.

Electrochemical sensing methods involve various electroanalytical techniques such as cyclic voltammetry (CV), differential pulse voltammetry (DPV), square wave voltammetry (SWV), linear sweep voltammetry (LSV), and amperometry for the determination of analyte. The electrochemical techniques involve three-electrode assembly such as working electrode, reference electrode, and counter electrode. Various kinds of working electrodes such as glassy carbon electrode (GCE), fluorine-doped tin oxide (FTO), indium tin oxide (ITO), carbon paste electrode (CPE), graphite electrode (GE), or nickel foam (NF) were used for electrochemical sensing applications. In contrast, silver/silver chloride (Ag/AgCl) and platinum (Pt) wire are used as reference and counter electrode, respectively. The modified working electrode interacts with the analyte, whereas reference electrode maintains the constant potential and counter electrode completes the circuit. The bare working electrodes such as GCE do not have sufficient electrochemically active surface for electrochemical reactions. Thus, bare surface of the working electrodes needs to be modified with electrocatalysts for the fabrication of electrochemical sensors. The working electrode can be modified by using drop-casting, electrodeposition, spin coating, and layer-by-layer assembly. Drop-casting method is widely used for the fabrication of electrodes for electrochemical sensing applications. The electrode fabrication for HQ sensing has been described in Scheme 1a. Schematic representation of the three-electrode assembly system is displayed in Scheme 1b.

It can be observed that electrochemical detection of HQ involves reversible oxidation–reduction as shown in Scheme 1a. Two bonds (oxygen–hydrogen) of phenolic hydroxyl groups break down and HQ releases two electrons and two protons, which formed quinoid structure. The first step involves the transformation of HQ to quinone by releasing two protons, whereas the second step is a reversible process which reforms HQ by accepting the released protons. The electrochemical sensing performance of the HQ sensor can be evaluated in terms of limit of detection (LOD) and sensitivity, which can be calculated by using the formula given below.

LOD = 3 × σ_e_/slope (1)

σ_e_ is standard deviation or standard error.

Sensitivity = Slope/Area of the working electrode(2)

In this section, we have compiled the progress in the fabrication of various electrode materials for electrochemical sensing of HQ.

### 2.1. Metal-Oxide-Based HQ Sensors

**Figure 1:**
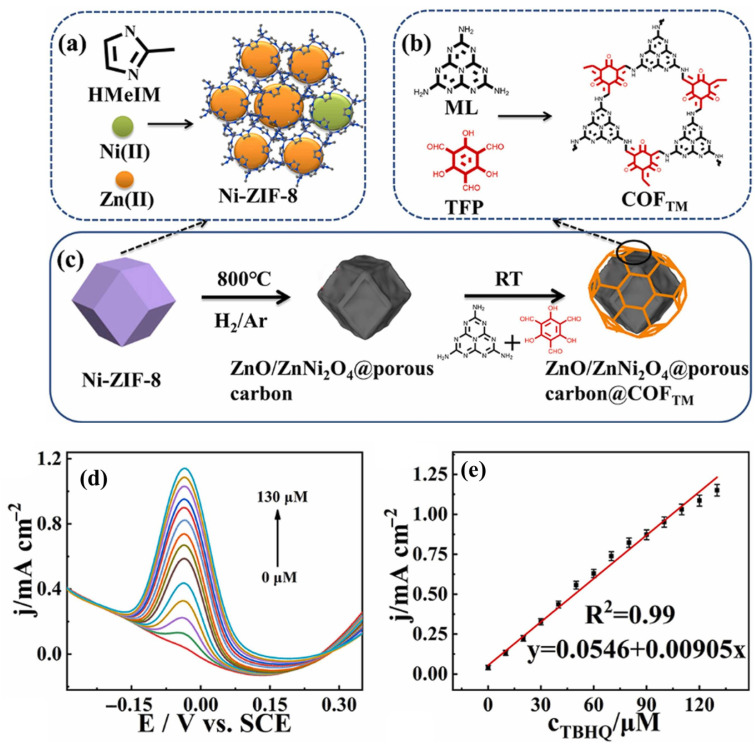
Schematic graph represents the fabrication of (**a**) Ni-ZIF-8, (**b**) COF_TM_, and (**c**) ZnO/ZnNi_2_O_4_@porous carbon@COF_TM_. (**d**) DPV curves of the fabricated TBHQ sensor with different concentrations and (**e**) corresponding calibration curve. Reproduced with permission [21].

As per the previous studies, it was observed that metal oxides are promising and stable electrode materials for sensing applications. In this context, a tert-butyl HQ (TBHQ) sensor was developed by preparing zinc oxide/zinc nickel oxide/porous carbon (PC)/covalent organic framework (ZnO/ZnNi_2_O_4_/PC@COF) as electrocatalyst [21]. Firstly, nickel-doped zeolite imidazolium framework-8 (Ni doped ZIF-8) was adopted as pyrolysis precursor for the formation of PC-encapsulated ZnO/ZnNi_2_O_4_ composite, which demonstrated a high surface area of 1130 m^2^/g. Secondly, COF_TM_ was successfully grown on ZnO/ZnNi_2_O_4_/PC composite and obtained ZnO/ZnNi_2_O_4_/PC@COF was characterized by sophisticated techniques, including X-ray photoelectron spectroscopy, transmission electron microscopy, and X-ray diffraction (XPS, TEM, and XRD). The fabrication process for Ni-ZIF-8, COF_TM_, and ZnO/ZnNi_2_O_4_@porous carbon@COF_TM_ is depicted in Figure 1a, Figure 1b, and Figure 1c, respectively.

**Figure 2:**
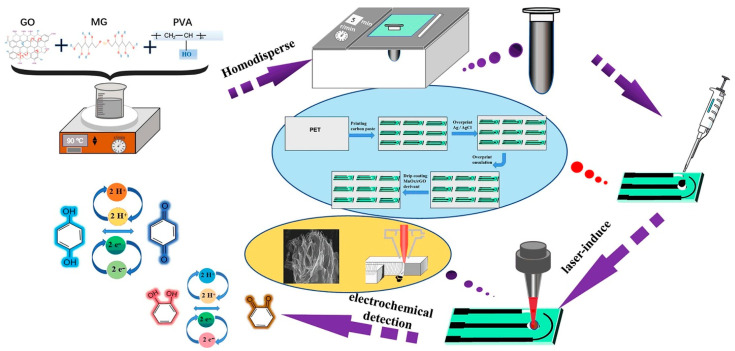
Pictorial view of the fabrication of MnO_x_/rGO/SPE. Reproduced with permission [32].

After formation of the electrode material (ZnO/ZnNi_2_O_4_/PC@COF), an HQ sensor was developed and its electrochemical sensing properties for TBHQ detection were performed using DPV technique. The obtained DPV curves of the modified electrode in the presence of various concentrations of HQ are shown in Figure 1d. The linear calibration plot between the peak current responses versus concentration of TBHQ is shown in Figure 1e. The current response linearly increases with increasing concentration of TBHQ. This modified GCE delivered LOD of 15.95 nM and wide linear range (LR) of 47.85 nM to 130 µM for TBHQ detection under optimized conditions. This sensor was also selective for TBHQ detection in the presence of various interfering substances. Zn@ZnO core shell structure was synthesized via pulse laser ablation method [22]. The presence of Zn in the Zn@ZnO core shell was authenticated by XRD with the presence of (100) and (101) diffraction planes, which corresponds to the JCPDS number 00-004-0831. The scanning electron microscopy (SEM)-based analysis revealed that Zn@ZnO has distorted spherical-shaped surface morphology. The energy-dispersive X-ray spectroscopy (XPS) confirmed the elemental composition and indicated the acceptable purity of the Zn@ZnO core shell. Electrochemical investigations revealed that Zn@ZnO/GCE has higher catalytic activities for HQ detection compared to the ZnO/GCE, which may be ascribed to the synergism in the Zn@ZnO core shell. As per the CV studies, it was observed that detection of HQ involves a diffusion-controlled process. The LOD, sensitivity, and LR of 0.10443 µM, 0.5673 μA μM^−1^ cm^−2^, and 10 µM to 90 µM were observed for HQ sensing using Zn@ZnO/GCE via CV. A temperature-controlled (On–Off) electrochemical sensor was also developed for the detection of HQ by employing poly (N-vinylcaprolactam) (PNVCL), reduced graphene oxide (rGO)@gold (Au), and monoclinic bismuth metavanadate (m-BiVO_4_)-based composite as sensing material [23]. The rGO@Au/PNVCL/m-BiVO_4_/GCE exhibits decent electrical conductivity and LOD of 600 nM was achieved by using simple linear sweep voltammetry (LSV) method. This electrode also has the potential to detect HQ with LR of 2 μM to 152 μM. The presence of the interfering substances did not affect the peak current response, which revealed the decent resistance of the proposed electrode for the interfering substances. Thus, it can be concluded that rGO@Au/PNVCL/m-BiVO_4_/GCE is selective for the determination of HQ in presence of interfering substances such as Pb^2+^, Mg^2+^, K^+^, Co^2+^, Na^+^, tartaric acid, glucose, urea, salicylic acid, NH_4_^2+^, and ethanol. Nitrogen (N)-doped rGO was incorporated with copper oxide (CuO) for the formation of N-rGO/CuO composite [24]. The obtained composite was further combined with ionic liquid modified with carbon paste electrode (ILCPE) for the sensing of HQ. The constructed electrode shows LOD of 250 nM and LR of 1 µM to 600 µM, with reasonable recovery of HQ in river and tap water samples. The modified CPE exhibited a selective nature for HQ sensing, which may be attributed to the synergy effects and improved electrocatalytic properties of the composite material. A cost-effective and sensitive electrochemical sensor was developed for the monitoring of TBHQ (2-(1, 1-dimethylethyl)-1, 4-benzenediol) by using porous cobalt oxide nanorods (Co_3_O_4_ NRs (embellished chemically oxidized carbon black (CB)) as electrode material [25]. The Co_3_O_4_ NRs/FCB (functionalized CB)-modified screen-printed carbon electrode (SPCE) showed LOD of 1 nM, and LR of 0.12 µM to 62.2 µM at pH 7.0 via DPV method. This modified SPCE also has good anti-interfering properties and stability for the monitoring of TBHQ. In a previous research work [26], chicken feather-waste-derived carbon-based molybdenum oxide (MoO_3_) was synthesized by using hydrothermal and co-pyrolysis method. The keratinous sludge-biomass-derived carbon (KSC)/MoO_3_ composite was characterized by XRD, which revealed the presence of acceptable crystalline nature and phase purity. The MoO_3_@KSC-modified SPCE was utilized as HQ and catechol (CC) sensor. The optimized conditions showed LOD of 63 nM and LR of 5 µM to 176.8 µM for HQ monitoring. The presence of enhanced catalytic properties and conductivity improved the sensing performance (stability, selectivity, reproducibility, and recovery in real samples such as Love River, Taiwan) of the proposed HQ sensor. Dalkira et al. [27] also reported a novel electrode (poly (safranine) (PSF) redox polymer/multi-walled carbon nanotubes (MWCNTs)/CuO-modified pencil graphite electrode (PGE)) for the construction of HQ sensor. The electrochemical studies revealed that LOD of 300 nM and LR of 1.5 µM to 100 µM can be obtained for HQ detection using PSF_Ethaline_/MWCNT/CuO/PGE via DPV technique. Cobalt tungstate (CoWO_4_; CoW) was also prepared by solvothermal method [28]. It was observed that synthesized CoW consists of uniform nanoplates with a size of 25 nm. The CoW-modified GCE was also electrochemically active with enhanced conductivity compared to the bare GCE. This electrode was further used as HQ sensor, which displayed LOD of 2.21 nM and LR of 0.02 µM to 0.1 µM and 0.12 µM to 0.32 µM with excellent selectivity. Manganese dioxide (MnO_2_) has promising electrochemical properties but suffers from low conductivity, which may limit the sensing behavior of the MnO_2_-modified electrodes. Thus, MnO_2_ NRs were combined with GO using hydrothermal method and characterized by SEM technique, which displayed the presence of MnO_2_ NRs on GO sheets [29]. The low transfer resistance of electrons and improved conductivity of the MnO_2_ NRs/GO composite-based electrode demonstrated LOD of 12 nM for HQ detection with LR of 0.5 µM to 300 µM with high selectivity. It can be understood that the presence of synergistic interactions improved the electrochemical activity of the MnO_2_ NRs/GO composite-based electrode. In another report [30], a novel electrode material (ZnO@MnO_2_/rGO) was synthesized by hydrothermal method. The presence of rGO may increase the surface area of the composite material and authors found that ZnO@MnO_2_/rGO-modified electrode has low charge transfer resistance (R_ct_) value compared to the bare GCE. The obtained ZnO@MnO_2_/rGO shows improved conductivity, which may be ascribed due to the presence of rGO. It was mentioned that ZnO@MnO_2_/rGO/GCE has the ability to detect the HQ with LOD of 1.2 nM and two LR of 0.008 µM to 10 µM and 10 µM to 320 µM with high selectivity. Co-precipitation method was adopted for the formation of Zn-doped CuO NPs [31]. Furthermore, Direct Yellow 11 (DY 11) was electro-polymerized on the surface of the Zn-doped CuO NPs-modified CPE via CV technique. This fabricated PolyDy11/Zn/CuO-modified electrode shows LOD of 7 µM LR of 10 µM to 90 µM with recovery of 90% to 94% in tap water sample for HQ detection. The modified CPE involved adsorption-controlled process for the sensing of HQ. Liu et al. [32] reported simultaneous detection of HQ and CC by utilizing MnO_x_/rGO-modified screen-printed electrode (SPE) via DPV technique. The fabrication of MnO_x_/rGO/SPE is explained in Figure 2.

Laser-induced one-pot synthesis method was used for the preparation of the electrode material. During the preparation of the composite material, initially, carboxyl groups on the surface of GO adsorb manganese ions and multivalent Mn ions were uniformly distributed and attached with rGO surface after laser treatment. It was also found that MnO_x_/rGO-modified SPE has enhanced electrical conductivity and it can detect HQ with LOD of 388 nM and LR of 20 µM to 300 µM. The presence of synergistic interactions improved stability, repeatability, and reproducibility of the modified SPE for HQ detection. Cerium tungstate/rGO (CeW@rGO) composite was obtained by hydrothermal treatment and its formation was confirmed by XRD and XPS [33]. The role of the binder for the stability of electrocatalyst was optimized. The synthesized CeW@rGO was carefully deposited on the surface of the GCE and its electrochemical activity of the modified GCE was evaluated by using CV and EIS techniques. The EIS studied revealed that CeW@rGO-modified GCE has decent electrical conductivity and can be used for HQ sensing application. The effects of pH and surfactant were also optimized to enhance the sensing performance of the proposed electrode for HQ detection. The CeW-6@rGO-modified GCE demonstrated LOD of 9.64 nM, LR of 0.1 to 115 µM, good reusability, high selectivity, decent long-term stability, and acceptable recovery in real water samples. This indicates that the present developed electrochemical sensor is a promising candidate for commercialization. Nam et al. [34] also successfully prepared manganese stannate/functionalized CB (Mn_2_SnO_4_/f-CB) composite and XRD analysis suggested the poor crystalline nature of the resulting composite material. The SEM analysis revealed that Mn_2_SnO_4_ nanocubes are present with f-CB, while EDX analysis confirmed the presence of Sn, Mn, and C elements in the prepared Mn_2_SnO_4_/f-CB composite. The Mn_2_SnO_4_/f-CB/SPCE shows that HQ can be determined by using LSV technique with LOD of 7 nM, sensitivity of 1.2903 µA µM^−1^ cm^−2^, LR of 0.005 µM to 70.80 µM, high selectivity, repeatability, stability, and acceptable recovery in pharmaceutical whitening sample. This work really exhibited interesting results with LOD in nM and recovery in pharmaceutical cream sample; therefore, it can be considered an efficient HQ sensor for practical applications. The synthesis of metal oxides using food fruit peels has attracted the scientific community due to the utilization of fruit waste. Thus, dried banana peels and dried orange peels were used for the preparation of bismuth oxide (Bi_2_O_3_) NPs and silica oxide (SiO_2_) NPs [35]. The fabricated composite of Bi_2_O_3_ NPs and SiO_2_ NPs was used as a sensing layer and combined with CPE for the monitoring of HQ. The SiO_2_/Bi_2_O_3_/CPE displayed good electrochemical activity for HQ detection in Nile River, bottle water, and tap water samples. It was found that an excellent LOD of 0.75 nM can be achieved for the determination of HQ by using DPV technique. The repeatability was checked by recording six consecutive DPV curves, which demonstrates decent repeatability with relative standard deviation (RSD) of 1.62%. This sensor was also found to be stable for up to 60 days and displayed good selective nature for HQ sensing. Gallium oxide (Ga_2_O_3_)-incorporated ZnO was fabricated using a simple chemical method [36]. The Ga_2_O_3_.rGO/GCE exhibited low Rct value, which suggested the presence of improved electrical conductivity compared to the Ga_2_O_3_ or rGO modified electrodes. The presence of improved electrical conductivity may boost electrocatalytic properties of the Ga_2_O_3_.rGO/GCE. Therefore, Ga_2_O_3_.rGO/GCE delivered good electrochemical performance in terms of LOD (63 nM), wide LR of 1 µM to 862 µM and 862 µM to 11,070 µM, sensitivity (1.0229 and 0.1408 μAµM^−1^ cm^−2^), recovery (96.1% to 99.1%) in tap water, stability, repeatability, reproducibility, and selectivity in the presence of various interfering substances. This performance of the Ga_2_O_3_.rGO/GCE may be attributed to the presence of synergism in the composite material, which provides fast electron transportation and larger surface area for redox reactions. Bismuth tungstate (Bi_2_WO_6_) is one of the low-cost and less toxic materials and can be utilized as a sensing layer for the construction of electrochemical sensors. In this context, Bi_2_WO_6_ NPs were synthesized and deposited on the surface of GCE and EIS study revealed that Bi_2_WO_6_ has better electrocatalytic properties and electrical conductivity compared to the bare GCE [37]. Therefore, Bi_2_WO_6_/GCE was found to be a promising candidate for the construction of HQ sensor, which can detect HQ with promising recovery in ointment sample. In another reported study, it was observed that Cu-doped Co_3_O_4_ (Cu_0.4_Co_2.6_O_4_) may be one of the promising sensing layers for the determination of HQ using electrochemical methods [38]. Thus, Cu_0.4_Co_2.6_O_4_ was obtained using hydrothermal method followed by calcination treatment. The Cu_0.4_Co_2.6_O_4_-based electrode exhibits high selective nature for HQ monitoring in the presence of various interfering substances. In another report [39], nickel oxide (NiO) was adopted as sensing material and NiO-modified CPE displayed LOD of 1.87 µM, LR of 5 µM to 50 µM, and selectivity for the determination of HQ. The presence of decent electrochemical properties of the NiO may be the key point of the interesting electrochemical performance of the NiO/CPE. Strontium-based manganese oxide (SrMnO_3_) perovskite material was synthesized by simple co-precipitation method [40]. Furthermore, SrMnO_3_ was incorporated with graphitic carbon nitride (g-C_3_N_4_) using hydrothermal method and formation of the SrMnO_3_/g-C_3_N_4_ composite was confirmed by XRD and XPS analyses. The obtained SrMnO_3_/g-C_3_N_4_ composite was casted on the surface of the GCE and CV/DPV techniques were adopted to evaluate its electrochemical performance for HQ detection. The SrMnO_3_/g-C_3_N_4_/GCE indicated that HQ can be detected with LOD of 6.32 µM and LR of 1 µM to 600 µM using DPV technique. It was proposed that the presence of improved active surface sites and synergism in the prepared composite material were the key points for the enhanced sensing performance of the above-mentioned HQ sensor. The Bi_2_O_3_/graphene ink-based electrode was also explored for the sensing of HQ, which displayed reasonably good selectivity [41]. In another study [42], zinc ferrite (ZnFe_2_O_4_)@f-carbon nanofiber (f-CNF) composite was obtained using sonochemical treatment. The SEM analysis confirmed the presence of the ZnFe_2_O_4_ NPs with f-CNF. The ZnFe_2_O_4_@f-CNF/GCE was utilized as HQ sensor, which displayed LOD of 26 nM, LR of 0.2 µM to 4013 µM, and real-time detection of HQ in cake and edible oil samples. It was also observed that the proposed HQ sensor is selective for the monitoring of HQ and presence of interfering substances such as caffeic acid, fructose, ascorbic acid, citric acid, uric acid, tryptophan, roxarsone, zeraalone, lactose, glucose, KCl, sucrose, NaCl, and NaNO_3_ does not affect the performance of the HQ sensor.

### 2.2. Carbon-Derivatives-Based Sensing Materials for HQ Detection

**Figure 3:**
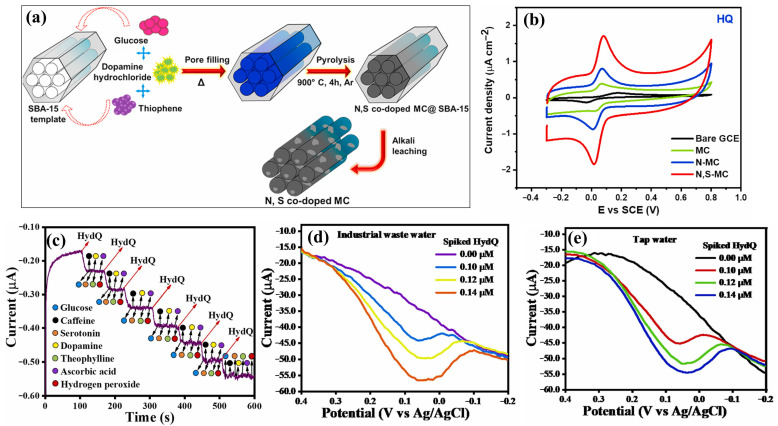
(**a**) Pictorial representation of the construction of N, S co-doped MC. (**b**) CV curves of different modified electrodes. Reproduced with permission [43]. (**c**) Selectivity test. Real sample study in (**d**) industrial wastewater and (**e**) tap water. Reproduced with permission [44].

In previous years, it was revealed from various reports that doping strategy may enhance electrochemical sensing performance of carbon-based materials by introducing or improving defective sites. Sivaraman et al. [43] found that nitrogen and sulfur (N or S)-doped mesoporous (NS-MC) carbon exhibits graphitic, pyridinic, and thiophenic defective sites. The synthesis of the proposed catalyst is shown in Figure 3a. The NS-MC-coated GCE shows improved electrocatalytic activity for the simultaneous determination of HQ using CV method (Figure 3b). Authors also adopted DPV technique and observed that current response increases for HQ detection with increasing concentration of HQ and it was found to be linear, as confirmed by linear calibration plot between the peak current response and concentration of the HQ. This electrode was also found highly selective for the simultaneous detection of HQ and CC. Authors also reported decent reproducibility, repeatability, and stability for HQ detection with LOD of 630 nM, LR of 2 µM to 16 µM and 16 µM to 40 µM, and sensitivities of 9.44 and 2.71 μA μM^−1^ cm^−2^. The presence of defective sites, enhanced electrical conductivity, and synergy between the doped hetero-atoms may be the key points for the enhanced sensitivity of the NS-MC-modified GCE for HQ detection. The 2D/2D heterojunction of g-C_3_N_4_ and hexagonal boron nitride (h-BN) was also developed for the electrochemical detection of HQ with improved sensitivity and selectivity (Figure 3c) [44]. The g-C_3_N_4_/h-BN-modified GCE displayed enhanced catalytic behavior and electrical conductivity; therefore, LOD of 9.23 nM with two LR of 0.02 µM to 0.08 µM and 0.09 µM to 0.17 µM was obtained for HQ detection. This indicates that g-C_3_N_4_/h-BN-modified GCE is a promising electrode for electrochemical sensing of HQ. Furthermore, practical ability of the proposed sensor for the detection of HQ in industrial water (Figure 3d) and tap water (Figure 3e) was also explored. The results showed acceptable recovery in the range of 97–99%. Thus, it is clear that the proposed g-C_3_N_4_/h-BN-modified GCE may be explored for real-time sensing of HQ.

Chen et al. [45] employed carbon aerogels-incorporated nickel@N-doped carbon core–shell nanoclusters for the development of HQ electrochemical sensor. Thus, authors prepared supramolecular hydrogels cross-linked Ni^2+^, chitosan (CS), and CNTs, which were adopted as a precursor for the fabrication of core–shell structured composite. The fabricated NCA_CNT_/Ni via pyrolysis method was coated on the surface of the GCE and utilized as HQ sensor. The NCA_CNT_/Ni/GCE delivered LOD of 360 nM, LR of 2 µM to 100 µM, and excellent selectivity in the presence of various interfering substances such as glucose, bisphenol A, Tannins, para-nitrophenol, K^+^, Na^+^, Al^3+^, etc., and high stability of two months. This electrochemical performance of the NCA_CNT_/Ni/GCE may be ascribed to enhanced hydrogen bonding and active sites induced by Ni nanoclusters in the prepared core–shell structure-based composite. Indium tin oxide (ITO) was modified with 3-aminopropyltriethoxysilane (APTES)/GO/Au NPs for the determination of acetaminophen, CC, and HQ [46]. The effects of pH and deposition time of electrode material were optimized and authors reported reasonable electrochemical performance of the ITO/APTES/GO/Au NPs electrode for the determination of HQ. Yang et al. [47] reported the synthesis of Ni-Zn-doped porous carbon (Ni-Zn@PC) for the construction of HQ sensor. The proposed electrode material was prepared by using ZIF-8 as template. It was observed that the presence of synergy effects between elemental Zn and PC enhances the electron transport properties of the Ni-Zn@PC-modified electrode. Thus, it was believed that Ni-Zn@PC may be a promising candidate for the sensing of HQ. The fabricated sensor delivered sensitivity of 180 μA μM^−1^ cm^−2^ and LR of 9.1 nM to 80 µM for HQ detection. The oxidative GO was also modified on the surface of GCE for the selective detection of HQ and CC [48]. The OGO/GCE shows that current response for the sensing of HQ linearly increases with increasing concentration of HQ. The OGO/GCE also shows good recovery of HQ in real samples, which suggests its potential for real-time sensing of CC and HQ. As per Rao et al. [49], boron (B) and N co-doping may create defects in the nanocarbon (NC)-based materials. The high density of B was introduced into the NC and prepared material can be labeled as BN-NC. The prepared BN-NC-based HQ sensor displayed LOD of 33.3 nM and LR of 0.099 µM to 43,340 µM using amperometric method. This improved electrochemical activity of the proposed HQ sensor may be ascribed to the generation of rich electroactive sites due to the co-doping of B and N in the NC. N and P co-doped glucose-derived carbon-coated CoP (G-CoP/N,P-C) nanowires (NWs) were explored as sensing layer for the monitoring of HQ and CC using voltametric method [50]. The G-CoP/N,P-C NWs were deposited on the surface of GCE and electrochemical investigations revealed that G-CoP/N,P-C NWs-coated GCE has excellent electrocatalytic properties with improved electrical conductivity. The CV- and DPV-based studies revealed that current response for redox peaks increases with increasing concentration of HQ and decent LOD of 180 nM with LR of 0.8 µM to 900 µM were obtained. This proposed HQ sensor was also selective for the monitoring of HQ and displayed acceptable recovery of HQ in lake water samples. Hu et al. [51] reported a novel electrocatalyst for electrochemical sensing of HQ and CC by using dual-template molecular imprinting technique. In this connection, dual-template molecularly imprinted polymer (MIP)/CNTs/GCE was developed. The presence of CC and HQ was detected by CV method followed by DPV technique. The anti-interference studies show that MIP/CNTs/GCE is selective towards the monitoring of HQ and CC in the presence of other interfering substances. Additionally, this electrode can detect HQ and CC in actual water samples. In another important study [52], mesoporous carbon hollow spheres (MCHSs)/ZIF-67-derived MCHSs/Co@N-CNT was obtained using ultrasonic-assisted method. The MCHSs/Co@N-CNT was casted on the surface of GCE and EIS results indicated that the proposed electrocatalyst has higher electrical conductivity compared to the bare GCE. Furthermore, CV analysis revealed that MCHSs/Co@N-CNT/GCE can detect HQ, resorcinol (RS), and CC simultaneously. Similarly, it was found that DPV technique is also promising for the simultaneous detection of HQ, RS, and CC. This electrode (MCHSs/Co@N-CNT/GCE) showed LOD of 270 nM with LR of 1 µM to 100 µM for the monitoring of HQ via DPV technique. Li et al. [53] found that covalent organic framework (COF)-integrated CNTs may be a suitable material for the construction of electrochemical sensors. In this context, authors prepared Co-porphyrin (TT-COF (Co)) and integrated it with N-doped CNTs and characterized by various techniques such as TEM, XRD, and XPS, which confirmed the formation of TT-COF(Co)/N-CNTs composite with decent purity. The TT-COF(Co)/N-CNTs composite-based electrode demonstrated LOD of 0.81 nM with satisfactory stability, selectivity, and real-time sensing in water samples. In another study [54], phenazine dye cresyl violet (CVio) was explored as a precursor for the fabrication of a novel electrochemical sensor using poly(cresyl violet)/MWCNTs as electrode material. Authors used novel solvents to optimize the performance of the proposed HQ sensor, which displayed good performance for the detection of HQ. Poly (quinine-co-itaconic acid)-incorporated rGO composite was synthesized and deposited on GCE surface [55]. The poly (quinine-*co*-itaconic acid)@rGO/GCE revealed that current response for the sensing of HQ linearly increases with increasing concentration of HQ. This electrode also indicated that HQ can be monitored with acceptable recovery of 96% to 96.6% in industrial water and 92% to 96.4% in river sample. The presence of synergistic interactions enhanced the electrochemical determination of HQ. The presence of synergism in the rGO and MWCNTs may exhibit enhanced electrocatalytic properties, electrical conductivity, and active sites for redox reactions. Therefore, rGO/MWCNTs-modified GCE demonstrated good reproducibility, selectivity, and stability for the determination of HQ [56]. The functionalization of CNTs has received great interest from researchers and previous study has indicated that carboxylated MWCNTs (c-MWCNTs)/poly(N,N-dimethylacrylamide) (PDEA) has excellent catalytic properties with improved surface area and interface interactions. Thus, PDEA/c-MWCNTs hydrogel film was fabricated for the sensing of HQ, which showed good recovery of HQ in tap water sample [57]. Jiao et al. [58] proposed that molybdenum (Mo)-, N-, and S-doped 3D porous carbon spheres can be an efficient electrocatalyst for electrochemical applications. Authors used polydopamine (PDA) as N source and template while thioacetamide (TAA) was used as S source and sodium molybdate as Mo source for the preparation of Mo-, N-, and S-doped interconnected porous carbon spheres (Mo,N,S-IPCS). The Mo,N,S-IPCS-coated GCE shows good electrochemical sensing of HQ with LOD of 47 nM and acceptable recovery of HQ in river water sample. Prussian blue nanocubes were electrochemically deposited on S-doped graphene for the selective detection of HQ [59]. It was observed that the proposed HQ sensor can monitor the HQ in real samples, which indicates its potential for real-time monitoring of HQ. In another work [60], Co NPs were anchored on N-doped carbon nanotube hollow spheres (HNC). The pyrolysis method was also used for the preparation of Co/HNC using ZIF-67@ZIF-8 as precursor. The Co/HNC-modified electrode revealed the presence of low charge transfer resistance (Rct) value, which indicates the existence of improved electrical conductivity compared to the bare GCE. The Co/HNC-modified GCE has the potential for the detection of HQ in detergents, lake/tap water, orange juice, and ointment samples. The enhanced electrochemical sensing performance may be attributed to the presence of larger surface area, improved active sites, porous microsphere structure, and shorter ion diffusion path. Zhou et al. [61] reported the formation of a hybrid composite of CoZn/CNTs/porous N-doped carbon and explored it as electrode material for the development of HQ electrochemical sensor. The CoZn/CNT@Por.N-C was drop casted on the surface of GCE and its electrochemical activity for HQ sensing was evaluated by employing CV and DPV techniques The reasonably good LOD of 2.11 μM was achieved using CoZn/CNT@Por.N-C/GCE. Authors stated that the presence of the abundant active sites and larger surface area enhanced its electrochemical activity for the monitoring of HQ. Rong et al. [62] also proposed the preparation of N-doped malic acid carbon quantum dots (N-MCQDs) using microwave and electrodeposition method. The N-MCQDs/GCE exhibits decent electrochemical performance for the sensing of HQ in terms of LOD, selectivity, reproducibility, and stability. The above-mentioned reports revealed that rGO and CNTs-based composites are promising electrode materials for the determination of HQ.

### 2.3. Metal-Sulfides-Based Materials for HQ Detection

**Figure 4:**
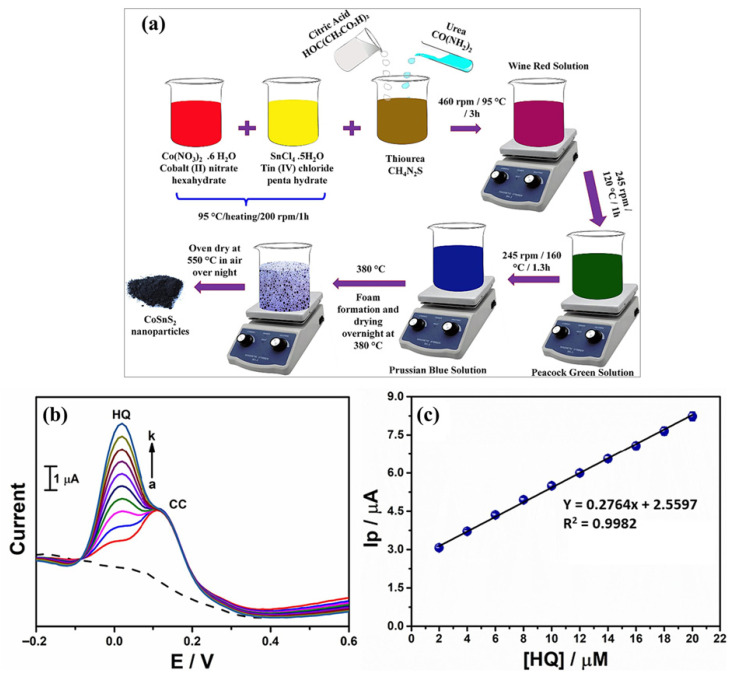
(**a**) Graphical representation of the preparation of CoSnS_2_. (**b**) DPV curves for the sensing of HQ and (**c**) corresponding calibration plot. Reproduced with permission [64].

Jahani et al. [63] reported the successful formation of lanthanum oxide (La_2_O_3_) NPs@snowflake-like copper sulfide (Cu_2_S) nanostructured composite. The obtained La_2_O_3_ NPs@SF-L Cu_2_S composite was characterized by XRD, which confirmed its formation with quadrilateral crystal system. The La_2_O_3_ NPs@SF-L Cu_2_S composite also has improved electrical conductivity and catalytic properties, which were revealed by CV and EIS analyses. The La_2_O_3_ NPs@SF-L Cu_2_S composite-modified electrode also detected HQ in tap water and mineral water samples with acceptable recovery of 95.4% to 102.5%. As per Ganesh et al. [64], it was observed that sol-gel-synthesized cobalt tin sulfide (CoSnS_2_) has nanocube-like surface morphology. Figure 4a shows a schematic graph for the synthesis of electrode material.

The presence of nanocube-like surface morphology and active sites enhanced the electrochemical sensing of HQ using CoSnS_2_/GCE. The fabricated CoSnS_2_/GCE also demonstrated excellent detection of HQ in the presence of CC (Figure 4b,c) with decent linearity. Therefore, LOD of 12.5 nM was observed for CoSnS_2_/GCE-based HQ sensor at pH of 7.4 via DPV method.

Jiang et al. [65] utilized microbial synthesis for the preparation of antimony sulfide (Sb_2_S_3_). Authors optimized the temperature for the synthesis of Sb_2_S_3_. The synthesized Sb_2_S_3_ at 700 °C exhibited improved electrical conductivity and electrocatalytic properties. Thus, Sb_2_S_3_-700-modified GCE detected HQ in tap water and river water samples with reasonably good recovery. In 2025, it was found that S-vacancy-rich CuS NPs-anchored N-doped CNF has larger electrochemically active surface area and more active sites [66]. Therefore, it may be a promising electrode modifier for the determination of HQ. Thus, CuS/N-CNFs-modified carbon paper electrode exhibited LOD of 293 nM, good selectivity, and repeatability. The above aforementioned results shows that metal sulfides may be the promising electrode materials for the construction of HQ sensors.

### 2.4. MXene-Based Materials for HQ Detection

**Figure 5:**
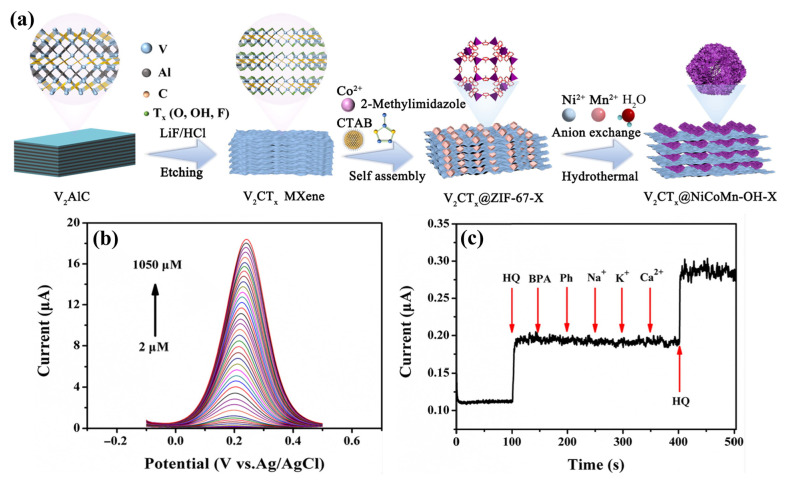
(**a**) Graphical view for the preparation of V_2_CT_x_@NiCoMn-OH-X. (**b**) DPV curves of the fabricated HQ sensor in different concentrations. (**c**) Selectivity test. Reproduced with permission [68].

MXene-based materials have been significantly adopted as conductive support and electrode materials for various electrochemical applications. In this connection, Ranjith et al. [67] reported the formation of 1D–2D composite of manganese molybdate (MnMoO_4_)-MXene. The proposed material was developed by employing hydrothermal and sonication methods. The obtained MnMoO_4_-MXene (titanium carbide (Ti_3_C_2_)) composite was deposited on GCE surface and CV, as well as DPV studies, which revealed that current response for the redox peaks linearly increases with increasing concentration of HQ. Ti_3_C_2_T_x_ MXene is a 2D layered metal carbide which possesses excellent metallic conductivity, larger surface area, and the presence of terminal groups such as –F, –OH, and –O that provides more active sites for electrochemical reactions towards the determination of HQ. The layered structure of Ti_3_C_2_T_x_ MXene exhibits improved conductivity, which may be provided due to the presence of conductive 2D planes with delocalized electrons which facilitate the electron transport process. It was observed that the proposed electrode involves diffusion-controlled process for the monitoring of HQ. The proposed electrode also demonstrated excellent selectivity and sensitivity for HQ detection. Yu et al. [68] explored the potential of 2D vanadium carbide (V_2_CT_x_) MXene for electrochemical sensing applications. The ZIF-67-derived nickel cobalt manganese hydroxides were combined with V_2_CT_x_ MXene (Figure 5a) and applied as electrode material for supercapacitor and HQ sensing applications. DPV studies show that current response increases with increasing HQ concentration (Figure 5b). The obtained 2D–3D structure (V_2_CT_x_@NiCoMn-OH-20) displayed good electrochemical activities for the monitoring of HQ using DPV and amperometry methods. It was also found that the proposed hybrid composite-based electrode exhibits excellent selectivity (Figure 5c) for the detection of HQ in the presence of various interfering substances via amperometry method. Han et al. [69] modified V_2_CT_x_ MXene via intercalation of CQDs and selenization strategies. The obtained heterostructure of VSe_2_@V_2_O_3_@V_2_CT_x_ MXene was deposited on GCE surface and explored as working electrode for the determination of HQ in three-electrode assembly. This proposed novel electrode showed good reproducibility, stability, selectivity, and repeatability for the sensing of HQ. The acceptable recovery of HQ in real water samples suggested its promising features for real-time sensing of HQ. It was observed that MXene-based materials not only exhibit improved electrical conductivity but also show enhanced electrocatalytic behavior for HQ sensing applications.

### 2.5. MOFs/ZIF/COF-Based Materials for HQ Detection

**Figure 6:**
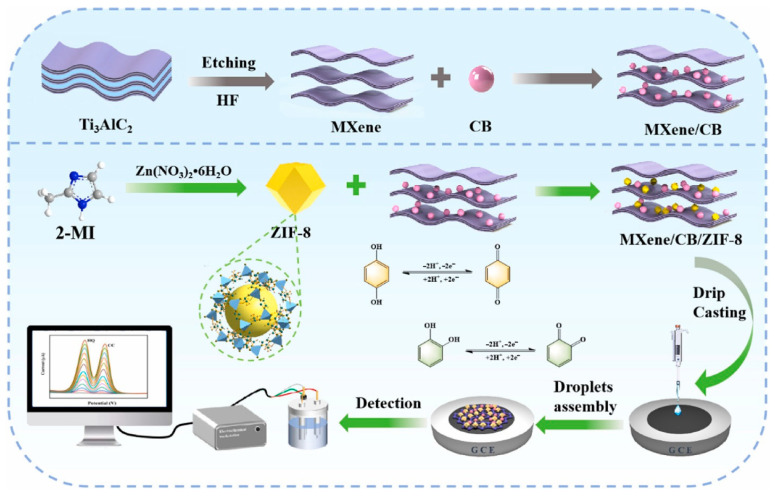
Schematic graphs for the fabrication of HQ sensor and its working mechanism. Reproduced with permission [78].

Metal organic frameworks (MOF) are known porous materials with larger surface area and exhibit promising characteristics for electrochemical applications. Shrimp-shell-derived porous carbon (SSPC) and Cu-melamine (Cu-Me) MOF composite was explored as sensing layer for the construction of HQ electrochemical sensor [70]. This electrode-material-based HQ sensor was able to achieve LOD of 1.83 µM, wide LR of 5 µM to 1800 µM, and high stability for HQ detection. The acceptable recovery of HQ in lake water was another advantage of the above-mentioned HQ sensor, which indicated its potential for real-time sensing applications. Liu et al. [71] prepared imine-bond-linked COF (TFPPy-B-COFD) using Schiff base reaction (1, 3, 6, 8-tetrakis (4-formylphenyl) pyrene and 4, 4′-diphenyldiamine) and integrated it with MWCNTs to develop the TFPPy-BD-COF/MWCNTs/GCE-based HQ sensor. This fabricated electrode has decent conductivity, improved catalytic properties, and reasonable porosity. These features make it suitable for the detection of HQ with enhanced sensitivity and selectivity. Sun et al. [72] also adopted simple strategies for the fabrication of TFPB-BD-COF (obtained by the condensation reaction of benzidine (BD) and 1, 3, 5-tris-(4-formylphenyl) benzene (TFPB)) and its composite with Pt NPs and amine (NH_2_) group functionalized MWCNTs. The TFPB-BD-COF/PtNPs/NH_2_-MWCNTs-modified GCE showed superior electrocatalytic activity for electrochemical detection of HQ, which may be ascribed to the synergistic effects (higher electrical conductivity, more active sites, and enhanced surface area) of the fabricated electrode. Therefore, interesting LOD of 22 nM, LR of 0.2 μM to 360 μM, decent selectivity, and acceptable recovery in river water, tap water, and domestic water samples were observed for the proposed HQ sensor. The MOF-derived materials also exhibit enhanced electrochemical performance for the sensing of HQ. Thus, simultaneous detection of CC and HQ was also reported by Wang et al. [73] using iron (Fe)- and N-doped carbon nanonets as sensing layer. Authors prepared leaf-shaped Fe-doped MOF, labeled as Fe-L-ZIF-8. The Fe-L-ZIF-8 was carbonized at higher temperature (under N atmosphere) to obtain the Fe- and N-doped carbon nanonets. This sensing material demonstrated good reproducibility, stability, selectivity, LOD of 170 nM, and LR of 0.50 to 80 μM. This may be due to the improved electrical conductivity of the Fe- and N-doped carbon nanonets compared to the Fe-L-ZIF-8. The Cu-based MOF (([Cu_4_{1,4-C_6_H_4_(COO)_2_}_3_(4,4′-bipy)_2_]_n_)) was prepared and adopted as a precursor for the fabrication of Cu-CuO@C composite [74]. The obtained Cu-CuO@C composite was coated on the surface of SPE and its electrochemical activity was determined by DPV method for the monitoring of HQ, which showed LOD of 390 nM and LR of 1 to 500 μM. Liao et al. [75] fabricated Ni/Zn-MOF by employing one-step solvothermal method. Furthermore, Ni NPs-supported porous Ni_3_ZnC_0.7_/Ni composite was obtained by pyrolysis approach under N atmosphere. The SEM analysis revealed that synthesized Ni_3_ZnC_0.7_/Ni composite at 750 °C has porous microsphere-like surface morphology. The presence of porosity in the prepared Ni_3_ZnC_0.7_/Ni composite may be attributed due to the pyrolysis of MOF structure. The Ni_3_ZnC_0.7_/Ni/GCE displayed improved stability and selectivity for the determination of HQ. It is expected that single-atom nanozymes (SANs) may improve the selectivity and sensitivity of the HQ electrochemical sensors due to the presence of active sites, high catalytic activity, and full atom utilization. Therefore, high-density Co-based SANs was anchored on activated MOF-derived porous carbon (Co-AcNC-3) using cascade anchoring method [76]. The Co-AcNC-3-based biosensor displayed wide LR of 4 μM to 300 μM, LOD of 34 nM, and selectivity. This enhanced electrochemical performance of the HQ biosensor may be attributed to the presence of high defectivity, higher surface area, abundant O-containing groups, and Co-N bonds. Calcium titanate (CaTiO_3_) perovskite/Zn-MOF/rGO composite was also explored as sensing layer for the fabrication of HQ sensor [77]. The presence of high conductivity of rGO and electrocatalytic properties of CaTiO_3_ enhanced the sensing performance of CaTiO_3_/Zn-MOF/rGO/GCE for HQ detection. Therefore, the proposed HQ sensor exhibited excellent selectivity, and stability, and real sample-based investigations suggested its potential for the monitoring of HQ in real-time sensing applications. In another study [78], ZIF-8/carbon black (CB)-modified MXene-based composite was also developed for electrochemical sensing applications. The obtained MXene/CB/ZIF-8 was deposited on GCE surface and it was adopted as working electrode for HQ sensing (Figure 6).

Typically, Ti_3_AlC_2_ MXene phase was obtained through etching process using HF as etching agent. Furthermore, Ti_3_AlC_2_ MXene/CB composite was obtained through ultrasonication approach. In another step, ZIF-8 was prepared, which was further combined with MXene/CB to form the MXene/CB/ZIF-8 composite via sonication treatment. Furthermore, MXene/CB/ZIF-8 was coated on GCE surface for the detection of HQ. The presence of the synergism in the prepared MXene/CB/ZIF-8 material improved the sensing of HQ in terms of selectivity, stability, and reproducibility. It is revealing that MXene-based materials may be promising electrocatalysts for the development of HQ sensors.

### 2.6. LDH/Polymers-Based Materials for HQ Detection

**Figure 7:**
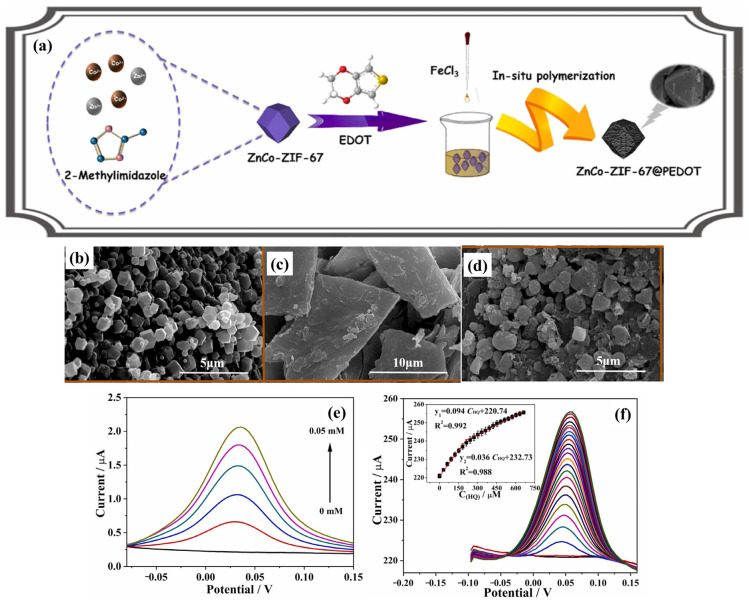
(**a**) Schematic representation of the synthesis of ZnCo-ZIF-67@PEDOT. SEM image of (**b**) ZnCo-ZIF-67, (**c**) PEDOT, and (**d**) ZnCo-ZIF-67@PEDOT. DPV curves of (**e**) ZnCo-ZIF-67 and (**f**) ZnCo-ZIF-67@PEDOT in different concentrations of HQ. Inset: calibration plot. Reproduced with permission [86].

The layered double hydroxide (LDH) materials have received extensive interest from the scientific community due to their excellent physicochemical and optoelectronic properties. The LDH-based materials exhibited impressive performance for various applications such as electrochemical sensors. LDH materials provide abundant active sites for redox reactions due to their positively charged metal hydroxide layers and interlayer anions. The presence of mixed-valence metal cations such as Ni^2+^/Ni^3+^ or Co^2+^/Co^3+^ may facilitate redox reactions, whereas high surface area and tunable composition allow for efficient electron transfer and ion exchange, which make LDHs materials highly efficient as electrode material for electrochemical applications. Liu et al. [79] developed O-vacancy-rich CoAl LDH-modified hydroxylated MWCNTs composite for the sensing of HQ. The fabricated OV-CoAl LDH/h-MWCNTs composite shows improved electrical conductivity and active sites for redox reactions. In addition, it was also found that the presence of the hydroxyl function groups in the h-MWCNTs facilitated the formation of hydrogen bonds with oxygen groups (present in the OV-CoAl LDH). Thus, a stable composite was formed, which displayed excellent selectivity and stability for the determination of HQ. He et al. [80] reported the fabrication of NiCoFe LDH nanoflowers (NFs) on hydrophilic carbon cloth (CC) for the simultaneous determination of HQ and CC. The proposed material was synthesized using benign hydrothermal method. The NiCoFe LDH NFs/CC has various advantages, such as 3D porous network structure, good electrocatalytic properties, electrical conductivity, and fast electron transportation. Therefore, NiCoFe LDH NFs/CC delivered interesting LOD of 150 nM and LR of 5 μM to 200 μM for HQ sensing. In other research work [81], CoFe LDH was adopted as sensing layer for the fabrication of HQ sensor, which displayed LOD of 1 nM selectivity, stability, and decent recovery in river water sample. Shan et al. [82] reported the fabrication of Prussian-blue-modified polyaniline (PB/PANI) nanoarrays under benign conditions. The synthesized composite PB/PANI shows improved conductivity and excellent electrochemical properties towards the sensing of HQ. The presence of larger surface area and active sites improved the selectivity and stability of the developed HQ sensor. Zuo et al. [83] developed flexible HQ sensor using Cu-CuO@MoO_x_-PEDOT (PEDOT = poly(3,4-ethylenedioxythiophene)) coated PET plastic electrode, which displayed excellent recovery of HQ in tap water samples via spike standard method. The tin oxide (SnO_2_)-based composite with PANI was proposed as sensing layer for the development of HQ sensor [84]. The obtained Co@SnO_2_/PANI composite was coated on GCE surface, which displayed decent LOD of 4.94 nM. Yu et al. [85] also reported the preparation of polypyrrole (PPy)-anchored NiCo bi-metal sulfide (NCS) composite for the electrochemical detection of HQ. The EIS studies revealed the presence of decent conductive properties in the fabricated PPy@NCS-5/GCE. In further studies, DPV analysis shows that redox peak current response increases with increasing concentration of HQ and LOD of 26 nM, LR of 0.5 to 1923 μM, and satisfactory recovery of HQ were obtained in oilfield wastewater sample. Sun et al. [86] also combined PEDOT with bimetallic MOF to improve the sensitivity and selectivity of the HQ sensor. The PEDOT polymer was epitaxially grown on ZnCo-ZIF-67 via in situ polymerization method, as demonstrated in Figure 7a. The SEM images for the prepared ZnCo-ZIF-67, PEDOT, and ZnCo-ZIF-67@PEDOT materials are displayed in Figure 7b, Figure 7c, and Figure 7d, respectively. It can be observed that ZnCo-ZIF-67 has dodecahedral-shaped surface morphology with smooth surface and they form a cluster-like structure. The PEDOT is consists of a compact thick sheet-like structure. In the case of ZnCo-ZIF-67@PEDOT, it was observed that incorporation of PEDOT with ZnCo-ZIF-67 reduced the agglomeration of ZnCo-ZIF-67 particles but did not affect its dodecahedral-like structure. However, prepared ZnCo-ZIF-67@PEDOT composite shows a rough surface compared to the pristine ZnCo-ZIF-67 material. Figure 7e shows that ZnCo-ZIF-67-based electrode has low catalytic properties for the determination of HQ, whereas ZnCo-ZIF-67@PEDOT-based electrode displayed high catalytic behavior for HQ detection, as shown in Figure 7f. Thus, ZnCo-ZIF-67@PEDOT-based electrode was capable of determining the HQ with LOD of 468 nM, two wide-range LR of 2 μM to 212 μM and 212 μM to 662 μM, and high selectivity. The presence of synergism in the hybrid composite may be the key factor for the enhanced performance of the proposed HQ sensor.

### 2.7. Other HQ Sensors

**Figure 8:**
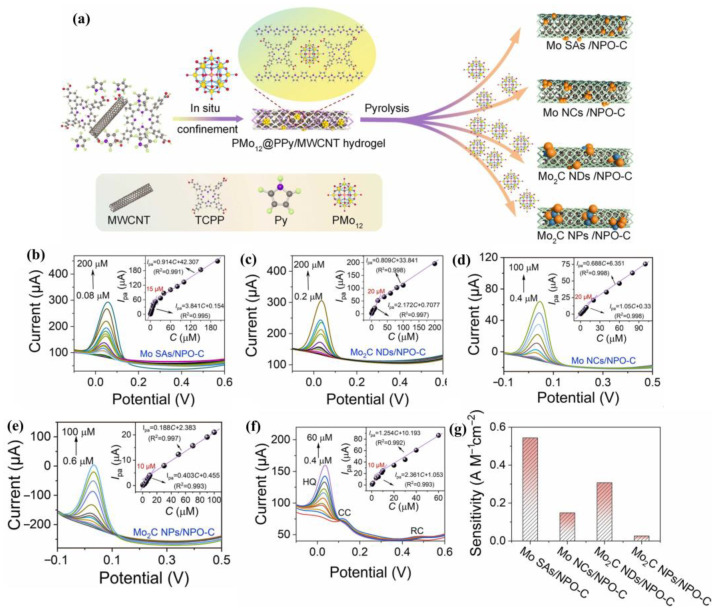
(**a**) Schematic representation of the preparation of Mo SAs/NPO-C, Mo NCs/NPO-C, Mo_2_C NDs/NPO-C, and Mo_2_C NPs/NPO-C. DPV curves of the (**b**) Mo SAs/NPO-C, (**c**) Mo NDs/NPO-C, (**d**) Mo NCs/NPO-C, (**e**) Mo_2_C NPs/NPO-C, and (**f**) DPV curves of Mo NCs/NPO-C for HQ detection in presence of CC and RC (resorcinol). Inset shows their corresponding linear calibration plots. (**g**) Sensitivity of different modified electrodes for HQ detection. Reproduced with permission [92].

Chuenjitt et al. [87] also proposed that poly (neutral red) (PNR) and porous graphene (P-Gr) may be a good choice for the construction of electrochemical sensor for environmental monitoring. Therefore, PNR/P-Gr was fabricated and deposited on GCE surface, which displayed higher electrical conductivity and high active surface area. Thus, PNR/P-Gr-modified electrode detected HQ in cream products with satisfactory recovery. The nano-flake graphite/bamboo-activated carbon (NFG/BAC) composite was also explored as HQ sensing material, which demonstrated LOD of 400 nM [88]. The increased active sites and surface area enhanced the sensing performance of the NFG/BAC-modified electrode for HQ detection. The acetylene black paste electrode (ABPE) was modified with salicylaldehyde-functionalized chitosan (S-CS) for the determination of HQ [89]. The electrochemical activity of the S-CS/ABPE was evaluated by CV and EIS analyses. Authors obtained LOD of 400 nM and LR of 1 µM to 80 µM using second-order derivative LSV technique. Fan et al. [90] also reported novel longquan lignite-derived hierarchical PC-based HQ sensor, which showed LOD of 160 nM. Another study also reported the construction of flexible HQ sensor using graphite/poly (butylene adipate-co-terephthalate) (PBAT) [91]. This proposed electrode displayed LOD of 1.01 µM and reasonable low cost (USD 0.17 per device) suggested its potential for practical applications. Cong et al. [92] reported that the preparation of Mo single atoms (SAs), Mo nanoparticles (NPs), Mo nanodots (NDs), and Mo nanoclusters (NCs) supported N, P, and O co-doped carbon (NPO-C) composites using supramolecular confinement pyrolysis method, as shown in Figure 8a. The prepared materials were used as HQ sensing materials. The DPV curves of the Mo SAs/NPO-C, Mo NDs/NPO-C, Mo NCs/NPO-C, and Mo_2_C NPs/NPO-C in the presence of various concentrations of HQ are displayed in Figure 8b, Figure 8c, Figure 8d, and Figure 8e, respectively. The Mo NCs/NPO-C-based electrode showed higher electrochemical performance for HQ detection compared to the other materials. Furthermore, DPV curves of the Mo NCs/NPO-C for HQ detection in the presence of CC and RC were also obtained and insignificant change was observed, which suggested its potential and high sensitivity for HQ detection. The sensitivity of various electrodes for HQ detection is shown in Figure 8g.

A disposable pencil graphite electrode (PGE) was also adopted as electrochemical sensor for the determination of HQ and CC, which displayed LOD of 850 nM for HQ detection with satisfactory recovery in tap water [93]. The sub-microsphere carbon (SMSC) was fabricated using *Sargassum coriifolium* seaweed and deposited on GCE surface [94]. The SMSC-modified GCE offers good accuracy, selectivity, and stability for the monitoring of HQ. Liu et al. [95] proposed the fabrication of HQ sensor using marine biomass shrimp-shell-derived PC (SSPC)/GCE as working electrode, which displayed excellent stability, selectivity, and satisfactory recovery of HQ in lake water sample. The longquan lignite ethanolysis residues were also used for the construction of PC-modified GCE-based HQ sensor [96]. The DPV measurements exhibit excellent stability, reproducibility, and selectivity for the monitoring of HQ using the above proposed electrode. Xue et al. [97] reported that mesoporous Ni (MNi) may be an efficient electrocatalyst for the development of electrochemical sensors. Thus, MNi-modified GCE shows improved LOD of 5.3 nM for HQ detection. Zhang et al. [98] prepared Au NPs-anchored layered yttrium hydroxide (LYH) using benign electrochemical method. The Au NPs/LYH-47/GCE shows that HQ can be detected with LOD of 200 nM and LR of 1 μM to 100 μM. It was observed that tunable morphological features of the LYH supports a great ability to influence the intrinsic electrocatalytic activity of Au NPs. In addition, the presence of synergism in the fabricated electrode material improved the sensing performance of the developed HQ sensor. An LOD of 56 nM was also reported for the determination of HQ using laser-induced Gr/Al NPs/PET nanocomposite [99]. Other work reported the construction of graphite screen-printed electrode (modified with EC-600JD Ketjen black (KB)) based HQ sensor, which displayed LOD of 2 nM [100]. A novel burnt carving pencil graphite electrode (BCPGE) was also adopted as electrochemical sensor, which delivered LOD of 118 nM using DPV technique [101]. Achache et al. [102] also reported sonogel-carbon (SNGC) modified with montmorillonite (MMT) as HQ sensor, which demonstrated decent electrochemical sensing performance for the determination of HQ in terms of selectivity and real sample recovery. The performance of the reported HQ sensors is summarized in Table 1.

The real sample analyses of HQ sensors are of great importance for practical applications. In various reports, standard addition method is widely used for the monitoring of HQ in real samples. In brief, DPV curves of the modified electrode were measured in real samples such as lake water or river water in the absence of HQ (only in blank) [103,104]. Subsequently, HQ spiked in the real sample and DPV curves of the modified electrodes were measured. This method is called standard addition method for the determination of HQ in real samples.

## 3. Challenges, Limitations, and Future Perspectives

Electrochemical sensors have been considered as next-generation electrochemical detection technologies for the monitoring of environmental pollutants. However, some limitations and remaining challenges restrict their potential application in real-time monitoring of HQ. As mentioned above, numerous electrode materials were used for the construction of HQ sensors, which exhibited remarkable performance but challenges exist, which are listed below.

- Metal oxides are cost-effective and stable electrode materials but have low conductivity, which can affect the electron transfer process and reduce the sensitivity of the fabricated electrodes for HQ detection.
- Carbon-based electrode materials such as CNTs, rGO, etc., offer high conductivity and larger surface area but agglomeration occurs, which can affect the selectivity.
- MXene-based materials are also considered as highly conducting materials but their synthesis process involves harsh etching conditions and low stability for long-term use.
- MOF and COF are well-known high-surface-area materials with decent functionality for electrochemical sensing applications but often show poor stability in aqueous environments.
- The polymer-based materials demonstrate improved conductivity and flexibility for the construction of electrochemical sensors but suffer from poor long-term stability.
- The selectivity of HQ in the presence of CC or RS may be compromised due to the isomeric properties of CC and RS.
- Real-time monitoring of HQ is still not convincing using standard addition methods, as mentioned in the manuscript.
- Commercial-scale fabrication of HQ sensors remained a key challenge.

Therefore, we believe that the above challenges need to be carefully studied in depth and addressed to explore the potential of HQ sensors for real-time sensing applications. We proposed the following few points, which can be considered for future studies.

- Simple and eco-friendly methods should be developed for the preparation of MXene materials.
- MXene/LDH materials or MXene/MOF materials need to be studied in detail and their mechanism for HQ sensing should be improved. Doping strategies may also be used to prepare the metal-doped MXene/MOF or MXene/LDH composites.
- Cost-effective, surface engineering, and scalable fabrication techniques need to be developed.
- Machine-learning-assisted sensors can be developed for HQ detection, which can be useful for the accurate detection of HQ.
- Wearable HQ sensors may be developed using flexible substrates.

## 4. Conclusions

It is concluded that HQ is one of the phenolic compounds which has negative impacts on human health and the environment. The release of HQ in ground water and the environment may cause various diseases to humans, affect aquatic life, and cause environmental pollution. Previous years have witnessed a rapid growth in the design and fabrication of numerous HQ electrochemical sensors. Electrochemical sensors are fast, simple, and highly sensitive for the determination of HQ compared to the conventional technique. The electrochemical detection performance of the sensors is largely affected by the physicochemical properties of the electrode modifiers. As discussed in this review article in Section 2, various electrode materials such as metal oxides, MXene, LDH, etc., are used for the construction of HQ sensors. The transition metal oxides and rGO-based composites show improved stability and selectivity for HQ detection. MXene-based materials are also promising new-generation electrode materials for the fabrication of HQ sensors.

## Figures and Tables

**Table 1:** Electrochemical sensing performance of the reported HQ sensors.

Electrocatalyst	LOD (µM)	Sensitivity	LR (µM)	Technique	Real Sample	References
Zn@ZnO core shell	0.10	0.5673 μA μM^−1^ cm^−2^	10–90	CV	-	[22]
*N*-rGO/CuO-ILCPE	0.25	-	1–600	DPV	Tap and river water	[24]
MoO_3_@KSC	0.063	-	5 to 176.8	DPV	River and lake water	[26]
CoWO_4_ nanoplates	0.00221	57.37 μA mM^−1^ cm^−2^	0.02–0.1 and 0.12–0.32	Amp	-	[28]
ZnO@MnO_2_-rGO	0.0012	-	0.008–10 and 10–320	DPV	Soyabean oil, tap water, orange juice, and river water	[30]
MnO_x_/rGO	0.388	-	20–300	DPV	Lake water	[32]
SiO_2_/Bi_2_O_3_	0.00075	-	0.005–5	DPV	River, tap, and drinking water	[35]
Bi_2_WO_6_	57	-	200–5000	SWV	Ointment	[37]
SrMnO_3_/g-CN	6.32	0.214 μA μM^−1^ cm^−2^	1–600	DPV	Wastewater and tap water	[40]
ZnFe_2_O_4_@f-CNF	0.026	-	0.2–4013	DPV	Edible oil and cake	[42]
g-CN/BN(1:1)	0.00923	-	0.02–0.08 and 009–0.17	DPV	Tap water and industrial wastewater	[44]
Oxidative graphene oxide (OGO)	0.114	-	-	DPV	Ground and wastewater	[48]
MIP/MWCNTs	3.1	-	10–100	DPV	River water	[51]
TT-COF(Co)/N-CNTs	0.81	-	0.003–300	DPV	Lake water	[53]
Poly (quinine-co-itaconic acid)@rGO	0.03	-	0.1–40	DPV	Industrial water, river water, and cream	[55]
Mo, N, S-IPCS	0.047	-	5–10,000	i-t	River water	[58]
Co/HNC	0.023	-	1–100	DPV	Lake water, tap water, orange juice, detergents, and ointment	[60]
N-MCQDs	0.18	-	1–500	DPV	Lake water	[62]
CoSnS_2_	0.0125	-	5–135	DPV	Tap water	[64]
CuS/N-CNFs	0.293	-	1–614	Amp	Landfill leachate, storm drain water, and textile mill wastewater	[66]
V_2_CT_x_@NiCoMn-OH	0.559	-	2–1050	DPV	Tap water	[68]
SSPC/Cu-Me	1.83	-	5–1800	DPV	Lake water	[70]
TFPB-BD-COF/PtNPs/NH_2_-MWCNTs	0.022	-	0.2–360	DPV	Tap water, river water, and sanitary sewage	[72]
Ni_3_ZnC_0.7_/Ni	0.14	-	0.3–100	DPV	Tap and river water	[75]
MXene/CB/ZIF-8	0.021	-	0.3–160	DPV	Upstream, midstream, and downstream water	[78]
NiCoFe-LDH NFs	0.15	-	5–200	DPV	Tap and lake water	[80]
Cu-CuO@MoO_x_-PEDOT/PET	0.221	-	5–870	i-t	Tap water	[83]
PPy@NCS-5	0.026	0.026 μA/μM	0.5–1923	DPV	Oilfield wastewater	[85]
NFG-BAC	0.4	-	2–1000	DPV	Lake water	[88]
PC/LLSP_800_	0.16	-	19.9–318.3	DPV	Environmental water sample	[90]
Pre-treated PGE	0.85	-	40–102	DPV	Tap water	[93]
SSPC	0.18	-	5–25 and 25–1200	DPV	Lake water	[95]
AuNPs/LYH-47	0.2		1–100	DPV	Tap and river water	[98]
Sparked KB	0.04	-	0.05–100	DPV	Tap water	[100]
MWNTs-IL-Gel	0.06	-	0.2–35	DPV	Tap water and mineral bottle water	[102]
CoP/Co_2_P@NC	0.02	-	0.05–80	DPV	River and lake water	[103]
CoFe_2_Se_4_/PCF-2	0.13	-	0.5–200	DPV	Lake water	[104]

## Abbreviations

Zn@ZnO	Zinc@zinc oxide
*N*-rGO/CuO-ILCPE	Nitrogen-reduced graphene oxide/copper oxide-ionic liquid modified carbon paste electrode
MoO_3_@KSC	Molybdenum oxide@keratinous sludge biomass derived carbon
CoWO_4_	cobalt tungstate
ZnO@MnO_2_-rGO	Zinc oxide@manganese dioxide-reduced graphene oxide
MnO_x_/rGO	Manganese oxide/reduced graphene oxide
SiO_2_/Bi_2_O_3_	Silicon dioxide/bismuth oxide
Bi_2_WO_6_	Bismuth tungstate
SrMnO_3_/g-CN	Strontium manganese oxide/graphitic carbon nitride
ZnFe_2_O_4_@f-CNF	Zinc ferrite@functionalized carbon nanofiber
g-CN/BN	Graphitic carbon nitride/boron nitride
OGO	Oxidative graphene oxide
MIP/MWCNTs	Molecularly imprinted polymer/multi-walled carbon nanotubes
TT-COF(Co)/N-CNTs	Cobalt porphyrin-based covalent organic framework/nitrogen doped carbon nanotubes
Mo, N, S-IPCS	Molybdenum, nitrogen, sulfur doped interconnected porous carbon spheres
Co/HNC	Cobalt/N doped carbon nanotube hollow spheres
N-MCQDs	Nitrogen doped malic acid carbon quantum dots
CoSnS_2_	Cobalt tin sulfide
CuS/N-CNFs	Copper sulfide/nitrogen doped carbon nanofibrers
V_2_CT_x_@NiCoMn-OH	Vanadium carbide MXene@nickel-cobalt-manganese hydroxide
SSPC/Cu-Me	Shrimp shell-derived porous carbon/copper-melamine MOF
TFPB	1, 3, 5-tris-(4-formylphenyl) benzene
BD-COF	Benzidine (BD) based covalent organic framework
PtNPs	Platinum nanoparticles
NH_2_-MWCNTs	Amine functionalized multi-walled carbon nanotubes
Ni_3_ZnC_0.7_/Ni	Ni nanoparticle-supported porous microsphere composites
CB/ZIF-8	Carbon black/zeolite imidazolium framework-8
NiCoFe-LDH NFs	Nickel cobalt iron-layered double hydroxide nanoflowers
CC	carbon cloth
Cu-CuO@MoO_x_	Copper/copper oxide@molybdenum oxide
PEDOT	Poly(3,4-ethylenedioxythiophene)
PET	Polyethylene terephthalate
PPy@NCS-5	Polypyrrole anchored NiCo bi-metal sulfide
GCE	glassy carbon electrode
NFG-BAC	Nano-flake graphite and bamboo activated carbon composites
PC/LLSP_800_	Porous carbon/longquan lignite soluble portion
PGE	Pencil graphite electrode
AuNPs/LYH-47	Gold nanoparticles anchored layered yttrium hydroxide
KB	Ketjen black
IL	Ionic liquid
CoP/Co_2_P@NC	Carbon-encapsulated cobalt phosphide nanoparticles and N doped carbon
CoFe_2_Se_4_	Cobalt iron selenide
PCF	Porous carbon nanofibers
CV	cyclic voltammetry
DPV	differential pulse voltammetry
SWV	square wave voltammetry
Amp	amperometry
